# Engineering the Future of Heart Failure Therapeutics: Integrating 3D Printing, Silicone Molding, and Translational Development for Implantable Cardiac Devices

**DOI:** 10.3390/bioengineering13020192

**Published:** 2026-02-08

**Authors:** Carleigh Eagle, Aarti Desai, Michael Franklin, Robert Pooley, Elizabeth Johnson, Shawn Robinson, Mark Lopez, Rohan Goswami

**Affiliations:** 1 Department of Radiology, Mayo Clinic, Jacksonville, FL 32224, USA; eagle.carleigh@mayo.edu (C.E.);; 2 Division of Heart Failure and Transplantation, Mayo Clinic, Jacksonville, FL 32224, USA; desai.aarti@mayo.edu; 3 Simulation Center, Mayo Clinic, Jacksonville, FL 32224, USA; 4 Innovation Exchange, Mayo Clinic, Jacksonville, FL 32224, USA

**Keywords:** 3D printing, silicone molding, heart failure, simulation, material jetting, stereolithography, anatomical modeling

## Abstract

Three-dimensional (3D) anatomic modeling derived from high-resolution medical imaging, such as computed tomography (CT) and magnetic resonance imaging (MRI), has been increasingly adopted in preclinical testing and device development. This white paper describes a cardiac-specific workflow that integrates 3D printing and silicone molding for support device development and procedural simulation. Patient-derived computed tomography angiography data were segmented using FDA-cleared medical modeling software to isolate the left ventricular anatomy and were further processed in computer-aided design (CAD) to ensure accurate physiological wall thickness and structural fidelity. Material jetting 3D printing was performed on a Stratasys J750 using material distributions designed to mimic the mechanical properties of myocardium, thereby approximating myocardial compliance. In parallel, stereolithography apparatus molds were designed from the left ventricle CAD model to cast transparent, pliable left ventricular models in Sorta-Clear™ 18 silicone. The 3D-printed models preserved intricate morphological detail and were suitable for mechanical manipulation and device deployment studies, whereas silicone models offered tunable mechanical properties, transparency for visualization, and durability for repeated use. Together, these complementary modalities provided rapid manufacturing capability and application-relevant physical representation. Case-specific parameters, strengths, and limitations of both models in enhancing patient care and device testing are highlighted, with relevance to heart failure applications. Current knowledge gaps, workflow and integration challenges, and future opportunities are identified, positioning this work as a reference framework for continued innovation in anatomic modeling. Within the collaborative framework of Mayo Clinic’s Anatomic Modeling Unit and Simulation Center, this integrated modeling workflow demonstrates the value of multidisciplinary collaboration between engineers and clinicians. Clinically, these patient-specific left ventricular models may enable pre-procedural device sizing and positioning and may support simulation of mechanical circulatory support (MCS) deployment while identifying possible anatomic constraints prior to intervention. This workflow has direct applicability in advanced heart failure patients undergoing MCS support, such as the Impella axillary MCS device or the durable LVAD, with potential to reduce procedural uncertainty while reducing complications and improving peri-procedural outcomes. Additionally, these models also serve as high-accuracy educational tools, enabling trainees and multidisciplinary care teams to visualize and possibly rehearse procedural steps while gaining hands-on experience in a risk-free environment.

## 1. Background

Cardiovascular disease remains one of the leading causes of morbidity and mortality worldwide, with one person dying of heart disease or stroke every 34 s [1]. This drives the need for innovative therapeutic strategies. As the complexity of cardiac disease evolves, so too must our approaches to treatment and device development. Engineering the future of heart failure therapeutics requires a multidisciplinary convergence of advanced imaging, materials science, biomedical engineering, and clinical translation. By integrating emerging technologies such as 3D-printed biomimicking models, high-fidelity silicone molding, and rigorous benchtop simulation, researchers and clinicians are reshaping the design and testing pipelines for implantable cardiac devices. These innovations promise not only to improve precision and safety but also to accelerate the path from concept to clinical application.

Multiple additive manufacturing techniques, including extrusion-based printing, stereolithography (SLA), and material jetting, have been applied to medical modeling [2]. Among these, material jetting has become a commonly used approach for fabricating high-fidelity anatomy models due to its ability to integrate multiple materials and achieve fine spatial control over geometry and mechanical behavior [3]. In parallel, stereolithography is frequently employed to produce accurate, smooth-surfaced molds suitable for downstream manufacturing processes, such as silicone casting [4,5]. The complementary use of material jetting for anatomically and mechanically representative digital models and SLA for mold fabrication enables the creation of transparent, compliant soft-tissue replicas while preserving anatomical detail, making this combined workflow particularly well-suited for cardiovascular simulation and device evaluation.

From a clinical perspective, the use of 3D printing is revolutionizing the diagnosis, planning, and treatment of cardiovascular diseases by enabling the creation of patient-specific anatomical models [6]. High-resolution imaging modalities, most commonly CT, generate accurate 3D digital reconstructions through a process called segmentation, which can then be 3D-printed to reflect a patient’s unique anatomy [7]. These models enhance surgical planning and clinical outcomes and can improve medical education by providing tactile learning tools for trainees [8]. As technology continues to evolve, 3D printing is paving the way for truly personalized medicine, allowing for interventions and therapies tailored precisely to the anatomical and physiological needs of individual patients.

Advancements in additive manufacturing have expanded the range of materials available for fabricating anatomically and mechanically realistic medical models, particularly in the context of replicating soft tissue [9]. The Stratasys J750 Digital Anatomy™ 3D printer (J750, Stratasys, Rehovot, Israel) employs material jetting technology to fabricate multi-material anatomical models with spatially varied mechanical properties [10]. This system, through the 3D printing slicer software GrabCAD Print (version 1.73), enables the deposition of photopolymer resins with precise control over composition and internal structure, allowing for the simulation of soft and hard biological tissues. It is capable of proprietary material presets, including configurations such as *Myocardium–Moderately Stiff*, *Subcutaneous Tissue*, and *Vessel Wall*, designed to approximate the compliance, durometer, and structural behavior of physiological tissues. Prior studies have demonstrated that the ‘Vessel Wall’ preset yields vascular models with clinically relevant distensibility across multiple vessel types [11]. These presets are optimized for medical modeling applications requiring clinically relevant mechanical fidelity, particularly in procedural planning, device testing, and surgical simulation.

Beyond predefined material presets, GrabCAD Print incorporates the Digital Anatomy Creator (DAC) feature, which enables advanced customization of material composition and internal architecture within a single anatomical model [12]. DAC allows users to generate custom digital materials by modifying photopolymer mixing ratios, outer layer thickness, background material composition, and infill architecture on a layer- and region-specific basis [13]. This voxel-based control provides a flexible framework for tuning mechanical behavior across complex geometries, enabling the creation of structures with spatially heterogeneous stiffness and compliance. While the present study relied on predefined material presets within GrabCAD Print, Digital Anatomy Creator is discussed here to provide context on the broader capabilities of material jetting-based anatomical modeling platforms.

3D-printed heart models derived from patient-specific imaging have rapidly transformed the testing and validation of medical devices. Unlike traditional animal models, 3D-printed models offer a highly accurate, customizable, and ethically responsible alternative that closely replicates human cardiac anatomy. By leveraging CT data and sophisticated software like Materialise Mimics Innovation Suite (version 25) (Leuven, Belgium), clinicians and engineers can create lifelike replicas of the heart’s internal structures for realistic device deployment simulations [14]. This approach not only enhances the precision and relevance of preclinical testing but also enables iterative design improvements, ultimately accelerating innovation and improving patient outcomes.

Furthermore, the choice of materials for advanced innovative medical devices or task trainers for medical education is by far the most important need that would make or break a custom design. Silicone materials must be clear, pliable, and impervious to liquids. Our institution at the Mayo Clinic in Florida, through a collaborative approach with the Multidisciplinary Simulation Center, is experienced in using a variety of silicone materials for multiple models in medical education devices. Within this paper, we describe the process of utilizing a clinician’s dilemma, a modeled heart, and an engineering powerhouse to build the solutions of tomorrow.

## 2. Development and Fabrication Pipeline for Left Ventricular Simulation Models

### 2.1. Development of the 3D CAD Model

The development of the 3D-printed model and corresponding silicone molds began with the selection of a single de-identified patient cardiac imaging study representing left ventricular cardiac anatomy. Cardiac computed tomography angiography (CTA) was selected as the imaging method. The CTA dataset was acquired using ECG-gated imaging reconstructed in diastole, with a slice thickness of 1 mm, and reconstructed using a standard soft-tissue reconstruction kernel (Br36f\2). All imaging data were fully de-identified prior to access, with protected health information removed in accordance with institutional policies. The investigators confirmed that a Mayo Clinic Institutional Review Board review was not required for this investigation. All patient-identifiable information was removed, and anonymization was maintained throughout the creation of the STL files.

**Figure 1:**
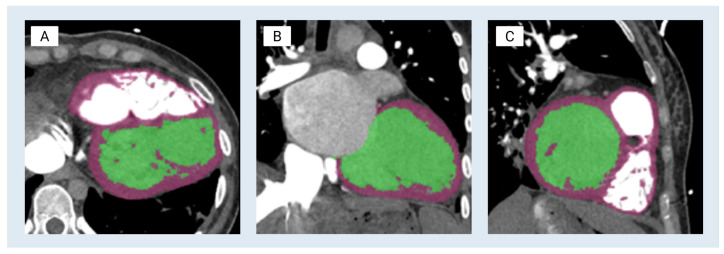
Segmentation process of modeling the left ventricle. The blood pools of the left ventricle and the myocardium are shown in green and purple, respectively. (**A**) Axial view—a short-axis slice through the left ventricle (classic round/oval LV cavity). (**B**) Coronal view—a long-axis view showing the LV from anterior to posterior. (**C**) Sagittal view—the orthogonal long-axis view, slicing left–right through the LV.

These scans were processed using Materialise Mimics (v.25), with segmentation performed by an experienced biomedical engineer under the guidance of a board-certified cardiothoracic radiologist to ensure anatomical accuracy. Initial segmentation of the left ventricular blood pool was performed using Hounsfield unit (HU) intensity-based thresholding appropriate for contrast-enhanced CTA (94–3071 HU) to generate a segmentation mask. Thresholding was limited to the region of interest, the left ventricle, through bounding boxes to eliminate irrelevant anatomy. A subsequent region grow operation was performed on the mask to denoise the initial threshold and refine chamber boundaries. This operation allows the user to select the shell of interest whilst removing extraneous structures that do not share a pixel face. The blood-pool mask was further trimmed to reflect only the left ventricle at the aortic and mitral valves (Figure 1B). The myocardium was subsequently segmented using a combination of thresholding (24–527 HU) and manual slice-by-slice refinement to generate a segmentation mask and correct for partial-volume effects and leakage at the mitral valve plane and interventricular septum. Segmented regions were color-coded for visualization: green for the left ventricle blood pool and purple for the myocardial tissue (Figure 1). Both masks were smoothed using the *Smooth Mask* tool to reduce pixelation and outliers. The complete segmentation workflow required approximately 3 h. Segmentation accuracy was assessed through comparison to the source CTA in axial, sagittal, and coronal planes, with confirmation of myocardial wall continuity and key anatomical landmarks prior to mesh export. Formal quantitative surface-to-image deviation metrics were not calculated in the present study and are an important area for future geometric validation in subsequent studies.

Following segmentation, the myocardium was exported as a 3D surface file and imported into Materialise 3-Matic (v.17) for mesh refinement and preparation. Mesh operations included controlled polygon reduction to optimize file size while maintaining geometric fidelity, and both global and localized smoothing to correct surface irregularities. External artifact distortions, such as stepped surfaces and jagged contours resulting from imaging noise, were manually smoothed, while selective internal smoothing operations were applied to achieve uniform wall surfaces and improve model integrity for downstream processing. The left and right ventricles were digitally separated to enable clearer chamber definition, and special attention was given to preserving the interventricular septum. Septal thickness was maintained to reflect physiologically representative proportions, particularly for the left ventricle, ensuring structural validity for device testing and silicone mold creation.

**Figure 2:**
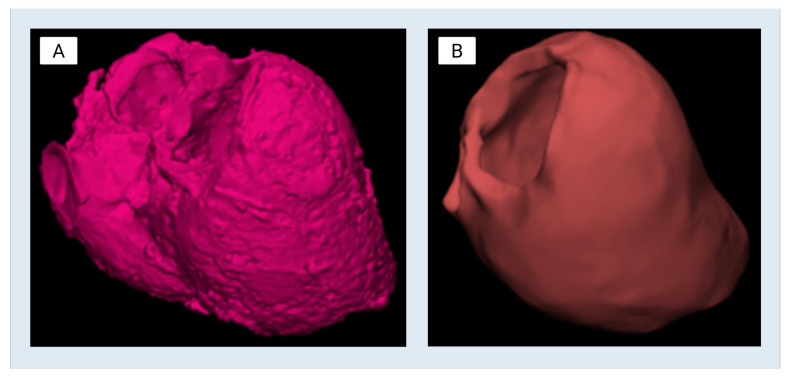
CAD modeling of the patient-derived left ventricle: (**A**) Full, unedited myocardium part prior to editing. (**B**) Left ventricle model after smoothing and elimination of the right ventricle.

**Figure 3:**
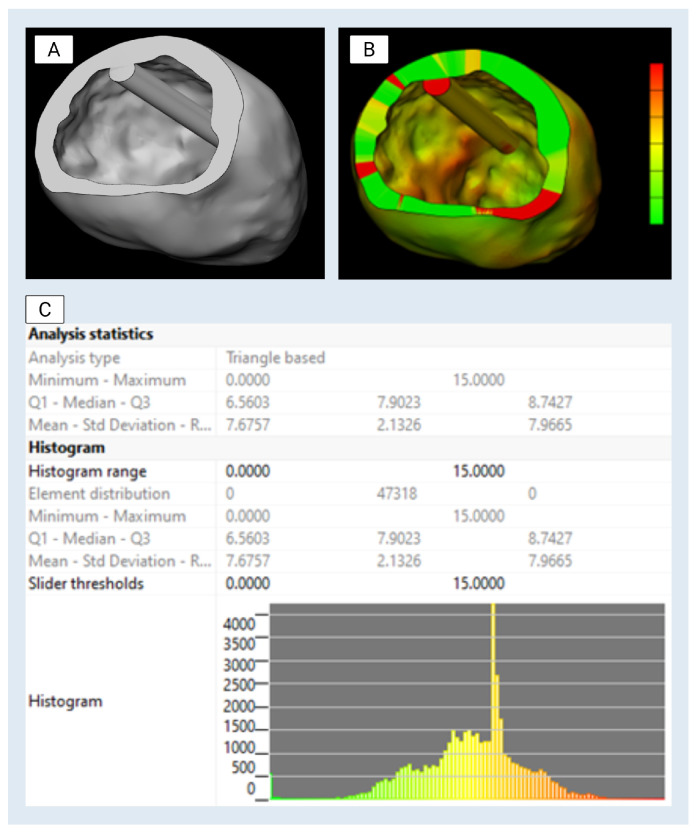
Final computer-aided design (CAD) model of the left ventricle with patient-derived wall thickness analysis: (**A**) Cross-sectional rendering of the left ventricle model, including a cylinder structure approximating papillary muscle location. (**B**) Wall thickness map overlaid on the model, with color coding representing local variations in thickness (green = low end, red = high end). (**C**) Histogram and statistical analysis of patient-derived wall thickness distribution, showing a majority of elements clustered around 7–8 mm. Color scale reference for wall thickness values (0–15 mm), corresponding to the model visualization in (**B**).

The finalized left ventricle (LV) CAD model was reimported into Materialise Mimics for qualitative verification by a board-certified cardiothoracic radiologist against the original CTA data (Figure 2). This step ensured anatomical consistency with patient-specific reference imaging. The model was then reviewed with the transplant cardiologist and annotated for design applications, including mold creation and internal structure simulation. To facilitate downstream use, multiple cut planes were established to expose relevant internal features. These included a “Center Cut” along the longitudinal axis of the ventricle, a “Top Cut” to enable superior access to the chamber, and the placement of a 70 mm cylindrical internal reference feature positioned within the ventricular cavity following papillary muscle smoothing (Figure 3). 70 mm sizing is in line with current clinical evidence for patients with dilated cardiomyopathy phenotype in terms of both positioning and imaging appropriateness. In our case, given the positioning of this structure and its non-anatomic/device-dependent nature [15], these design features were incorporated to enhance visualization, enable silicone molding, and accommodate device deployment studies.

While left ventricular wall thickness was entirely patient-derived, internal trabeculations and papillary muscle structures were intentionally smoothed during mesh refinement to simplify internal geometry and improve manufacturability, mold release, and device access for benchtop testing applications. To preserve a consistent internal spatial reference within the ventricular cavity following this abstraction, a cylindrical reference feature was introduced. This feature serves as a non-anatomical placeholder representing the approximate spatial region of the papillary muscle complex and is not intended to replicate native papillary muscle anatomy or dimensions.

The finalized CAD model of the left ventricle was evaluated for 3D printing readiness, including inspection for mesh integrity, watertightness, and the correction of any geometric or topological errors. Upon verification, the model was exported in STL format for additive manufacturing. A duplicate of the verified STL file was generated for use in downstream mold design and silicone casting workflows.

### 2.2. 3D Printing of the Left Ventricle Using Material Jetting

**Figure 4:**
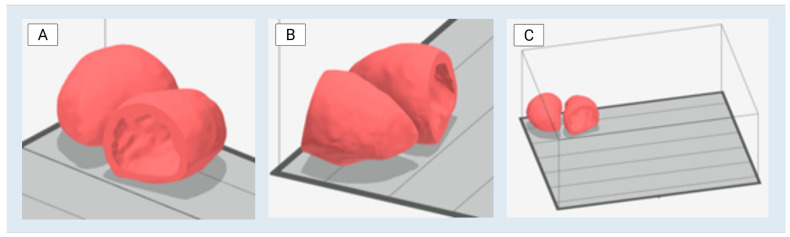
Left ventricle duplicate models in the Stratasys GrabCAD software environment, prepared for 3D printing on the Stratasys J750 3D printer. (**A**) fucses on the base of the heart—the open portion. (**B**) focuses on the apex of the heart—the conical portion. (**C**) Bird’s eye view.

The finalized left ventricle STL file was imported into Stratasys GrabCAD Print software and assessed for compatibility with the J750 3D printer (J750, Stratasys, Rehovot, Israel). The model was positioned and prepared according to the printer’s requirements, including support structure optimization and material preset selection. Two identical instances of the model were generated within the build tray to enable multiple prints for subsequent testing and validation procedures (Figure 4).

**Figure 5:**
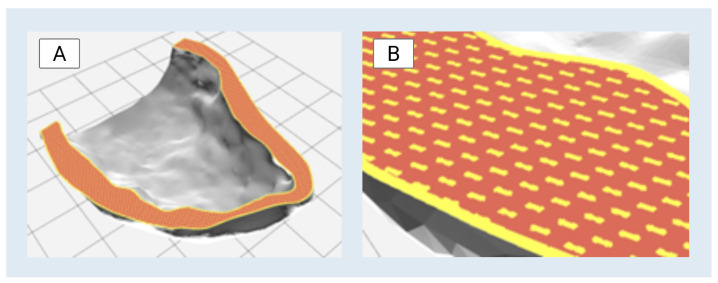
(**A**) Visualization of voxel-level material composition within GrabCAD Print’s Digital Anatomy Creator suite for the left ventricle model. (**B**) The selected “Structural Heart–Myocardium–Moderately Stiff” preset is shown with a gyroid-patterned infill of Agilus30™ Clear (orange) embedded within a TissueMatrix™ background (red) and encapsulated by a 0.4 mm solid outer shell of Agilus30™ Clear (yellow). This material configuration is designed to replicate myocardial mechanical behavior while maintaining print integrity and anatomical fidelity.

The Digital Anatomy preset library within GrabCAD Print was used to define the material configuration for the left ventricle model. The “Structural Heart–Myocardium–Moderately Stiff” preset was selected to simulate myocardial mechanical properties. This configuration included a gyroid-patterned infill composed of Agilus30™ Clear and a surrounding matrix of TissueMatrix™ material. To enhance structural integrity during handling and testing, the outermost shell was assigned a solid 0.4 mm layer of Agilus30™ Clear. A visual representation of this material configuration is shown in Figure 5.

The two identical left ventricle models were arranged and optimized on the build tray within GrabCAD Print according to printer-specific orientation and material flow preferences. A print time estimate was generated, and the 3D print was completed in approximately 14 h and 45 min. Upon completion, the models were carefully removed from the build platform and subjected to post-processing to eliminate residual support material.

Our protocol may differ, and each process should be individualized. We describe our process here. The initial post-processing involved manually removing bulk wax-like support structures using appropriate tooling. The models were then immersed in a 2% sodium hydroxide (NaOH, CAS 1310-73-2)-1% sodium metasilicate (Na_2_SiO_3_, CAS 6834-92-0) aqueous solution. The support material concentration in density of the solution was maintained at 1.030–1.040 ± 0.002 g/cm^3^ according to manufacturing best practices [16]. The temperature of the solution was not recorded, although the laboratory’s ambient temperature was maintained at 23 °C. The solution was maintained per manufacturer guidelines and disposed of per institutional waste disposal policies at saturation, density ≥ 1.045 ± 0.002 g/cm^3^. A 3 h agitation cycle on high speed was set; then, models were allowed to soak in the static solution for 8 h to facilitate the dissolution of remaining support material from internal and external surfaces.

Following chemical soaking, the models were rinsed thoroughly with pressurized filtered water (Nephros DSU-HUltrafilter) in the Balco Powerblast and allowed to air-dry for 24 h. No visible surface degradation or otherwise loss of structural integrity was observed following alkaline soaking. Models were confirmed dry upon physical inspection, including a visual transparency change from opaque to semi-transparent, witnessed by an experienced engineering technician. Post-processing was considered complete once all support residues had been removed, and the models were visually and mechanically verified for structural integrity. Based on qualitative inspection and handling, exposure to the solution and pressurized rinsing under the outlined conditions did not result in observable changes to the mechanical behavior of the printed materials.

**Figure 6:**
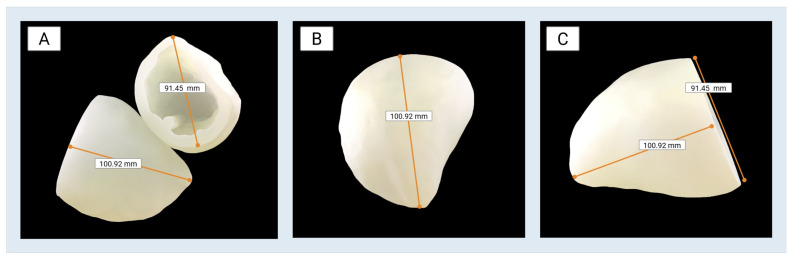
Final 3D-printed left ventricle models produced using the Stratasys J750 Digital Anatomy Printer: (**A**–**C**) Multiple views of the final, post-processed prints are shown to demonstrate external morphology, internal chamber detail, and surface quality achieved using the Structural Heart–Myocardium–Moderately Stiff material preset. The model has a base-to-apex length of 100.92 mm and a maximum external diameter of 91.45 mm. The semi-translucent appearance of the models is expected due to the use of Agilus30™ Clear and TissueMatrix™ materials and reflects the intended Digital Anatomy material configuration rather than incomplete post-processing.

The completed 3D-printed models of the left ventricle accurately preserved both external morphology and internal chamber detail, as defined by the selected Digital Anatomy material preset. The final prints demonstrated high structural integrity and visual fidelity, with consistent wall thickness and a compliant, tissue-like texture suitable for downstream benchtop testing. As shown in Figure 6, the models maintained smooth surface contours and anatomical geometry, enabling their use in physical simulations. These models served as physical representations of digital designs capable of mechanical manipulation for testing purposes.

### 2.3. 3D-Printed SLA Molds for Silicone Molding

To generate the mold geometry, the finalized left ventricle model was offset outward by 15 mm and 30 mm to create two concentric negative shells. These volumes were then sectioned along predefined “Top Cut” and “Center Cut” planes to support mold separation and silicone casting. Due to the anatomical complexity of the left ventricle, including deep cavities and undercut regions, the mold was designed as two separable halves. This two-part configuration was necessary to enable safe removal of the cured silicone model without risking deformation, tearing, or damage to intricate internal features. A single-piece mold would have impeded demolding and compromised the anatomical fidelity of the final cast.

The dual-part mold design facilitated uniform material flow, simplified silicone pouring and venting, and incorporated integrated alignment features for consistent mold closure. The final mold halves were exported. Each half conformed precisely to the surface geometry of the left ventricle to ensure high-fidelity reproduction in silicone.

**Figure 7:**
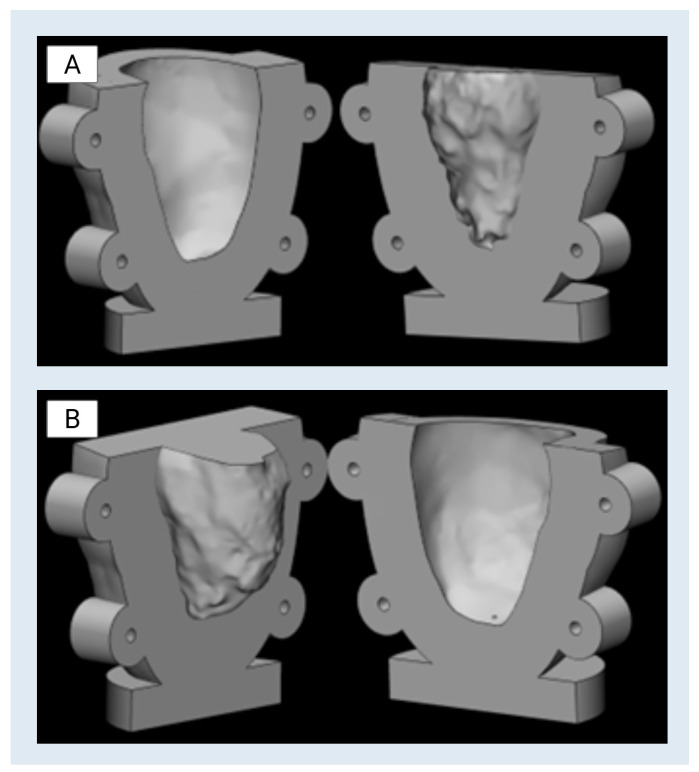
(**A**) CAD rendering of Mold Half 1, showing the internal cavity shaped by the left ventricular anatomy. (**B**) CAD rendering of Mold Half 2, showing the internal cavity shaped by the left ventricular anatomy.

Figure 7, shown above, displays the CAD renderings of the two-part molds used for casting the left ventricle. The mold housing incorporates thick outer walls and rounded corners to enhance durability, while the flanged edges with bolt holes allow for secure clamping under pressure. This design approach ensures the internal fidelity of the cast while also addressing the practical constraints of mold alignment, material flow, and post-cure demolding.

The finalized mold designs were exported as STL files and prepared for printing using PreForm software (version 3.27.1.) (Formlabs, Inc. Somerville, MA, USA). The molds were printed on a Formlabs Form 3BL stereolithography apparatus (SLA) 3D printer using White Resin V3, a rigid photopolymer well-suited for high-resolution molding applications. Each mold half was oriented to minimize the need for internal support within the cavity region, ensuring a smooth surface finish and dimensional accuracy. A layer height of 100 microns was selected to balance detail resolution with print time efficiency. After printing, the parts were washed in isopropyl alcohol (IPA) for 10 min to remove uncured resin and then post-cured under 405 nm light at 60 °C for 15 min using the Form Cure unit to achieve full mechanical properties.

Following post-curing, support structures were carefully clipped from the printed mold halves using flush cutters to prevent damage to the mold geometry, particularly near the internal cavity surfaces. Residual support contact points were then sanded using progressively finer grit sandpaper to ensure smooth mating surfaces and prevent leakage during silicone casting. Special attention was given to the interior mold cavities to preserve anatomical accuracy and surface integrity. After surface finishing, each mold half was visually inspected and manually aligned to verify proper registration of anatomical features and mold hardware interfaces. This inspection ensured dimensional consistency, sealing integrity, and overall readiness for casting workflows.

### 2.4. Silicone Molding of Left Ventricle

To assemble both two-part molds, petroleum jelly was applied on the edge of each side, and (4) 2 1/4” screws, nuts, and washers were inserted to keep both molds sealed. No additional surface coating was applied to the SLA-printed molds, as the native surface finish was sufficient for silicone casting.

For the silicone model creation, Sorta-Clear 18™ silicone rubber was prepared according to the manufacturer’s recommended mixing ratio of 1:1 (Part A:Part B by weight) [17]. The anterior mold half required 86 g of mixed silicone, and the posterior mold half required 68 g, resulting in a total mixed silicone mass of 154 g. Accordingly, 77 g of Part A and 77 g of Part B were thoroughly mixed and poured into the assembled ventricle molds. The silicone was allowed to cure for 24 h at room temperature.

For the silicone model assembly, pull apart both left ventricle molds to retrieve the silicone models. To glue both halves together with Sil-Poxy™, a silicone adhesive specifically formulated to bond RTV silicones [18]. This technique resulted in a strong, permanent bond between the mold halves without compromising anatomical detail or mold performance during casting.

## 3. Discussion

This work describes a translational approach to developing anatomically and mechanically representative left ventricular models through the integration of material jetting, SLA mold fabrication, and silicone casting. Moving beyond visualization toward functional modeling, this workflow aims to approximate the geometry and mechanical behavior of cardiac tissue. This is a step beyond the novel technologies of augmented reality, providing tactile and visual feedback not available in purely digital workflows. Mechanical and physiological relevance are discussed in terms of design intent and anticipated behavior; quantitative validation against biological tissue is identified as an important area for future work.

To conclude our process, the mold was utilized to define the properties of a novel device for myocardial recovery and stability. This process entailed the utilization of said left ventricular molds in the process of defining positioning, deployment, and subsequent testing within a sealed pressure chamber to define initial operational thresholds. Given the novelty of the device and the ongoing work in the clinical space, we are unable to provide specific technical details within the confines of this white paper.

### 3.1. Platform Optimization

The Stratasys GRABCAD platform is instrumental in bridging the gap between medical imaging and benchtop simulation. The use of the *Structural Heart–Myocardium–Moderately Stiff* preset enables rapid fabrication of anatomically representative models with tunable compliance and internal structure. However, while the preset offers intuitive customization and internal gyroid infill patterns intended to emulate cardiac tissue, its utility is limited by a lack of publicly available mechanical property data and direct quantitative comparison to biological myocardium.

In the absence of true mechanical characterization to directly quantify biofidelity of materials used, mechanical relevance is discussed in the context of material selection, design intent, and prior use of similar silicone formulations. While limited myocardial tissue properties have been reported in the literature, no quantitative evidence is claimed here between the fabricated models and native myocardium beyond the qualitative assessment of a clinician. Furthermore, variability in myocardial energetics and innate tissue properties cannot be truly replicated in silico, but are approximated as closely to physiologically capable as current technologies allow. The materials selected for both the 3D-printed model and the silicone cast model were chosen to enable controlled, repeatable mechanical behavior relative to the intended material properties of myocardial tissue. Establishing a comprehensive dataset that links digital anatomy material presets to physiological tissue properties remains a critical step for the broader adoption of these materials in preclinical device development and surgical planning.

To address this limitation, our workflow incorporates silicone molding as a complementary fabrication pathway, as it is closer to the commonly observed industry standard. By producing high-fidelity SLA show molds directly from patient-specific 3D data, the method enables the casting of transparent, compliant models using well-characterized silicone materials such as Sorta-Clear™ 18. The mold design, split into two separable halves, was essential to preserve fine internal geometries during demolding and reduce strain on delicate structures such as the papillary muscle interface and septal wall.

These workflow models are intended to support mechanical representation and allow for design-level control over bulk mechanical behavior through material selection and wall thickness modulation. The design parameters can be leveraged to account for pathological cardiac conditions in which myocardial mechanical properties vary spatially and temporally. Disease states such as left ventricular hypertrophy, myocardial fibrosis, ischemic stunning, and infiltrative cardiomyopathies are characterized by localized changes in stiffness, compliance, and wall thickness. Within the 3D printing workflow, such effects could be explored through regionally varied wall thicknesses, selective incorporation of softer or stiffer materials, or graded transitions between material regions to mimic heterogeneous myocardial remodeling. While the present discussion is focused on achieving anatomically accurate models with controlled bulk mechanical behavior, quantitative validation of regionally varying mechanics was outside the scope of this work.

### 3.2. Post-Printing Performance Analysis

In exploring methods for mold preparation, we evaluated the use of XTC-3D, an epoxy-based coating commonly applied to improve the surface finish on FDM-printed materials such as PLA, Tough PLA, and ABS. While effective in smoothing rough surfaces and sealing porous prints, XTC-3D was deemed unsuitable for our SLA-printed left ventricle molds due to its incompatibility with the already smooth surface finish of SLA resin and its tendency to discolor over time, which could interfere with visual inspection and long-term mold reuse. Additionally, we tested multiple bonding techniques for assembling the two mold halves. An initial attempt to seal the mold using Sorta-Clear™ 18 silicone as an adhesive was unsuccessful, as the material failed to cure fully when applied between large mating surfaces. The final and most effective method employed was Sil-Poxy™, a silicone adhesive specifically formulated to bond RTV silicones. This technique resulted in a strong, permanent bond between the mold halves without compromising anatomical detail or mold performance during casting. As a result, XTC-3D was excluded from the final workflow and is not recommended for SLA-printed molds in this application.

Together, the dual-modality approach, combining 3D printing for rapid anatomical modeling and silicone molding for functional replication, supports the development of a scalable innovation ecosystem. Within Mayo Clinic, this ecosystem is anchored by a collaborative infrastructure that brings together radiologists, engineers, simulation specialists, and clinicians. The result is an integrated pipeline capable of turning medical imaging into device-ready, physiologically inspired, and reproducible models.

Moving forward, expansion of this work will focus on mechanical benchmarking of printed and molded materials, validation against excised human tissue, and iterative feedback from end users, including interventional cardiologists, surgeons, and industry collaborators. Additionally, efforts to streamline access, reduce fabrication costs, and integrate these models into procedural training and regulatory workflows will be crucial for clinical implementation.

### 3.3. Clinical Translation

At our institution, the collaboration between the Division of Heart Failure and Transplantation, the Anatomic Modeling Unit (AMU), and the Multidisciplinary Simulation Center exemplifies a forward-thinking and innovative ecosystem. By combining biologically inspired 3D printing, custom silicone molding, and iterative simulation workflows, we are developing a streamlined pipeline that transforms clinical challenges into tangible solutions.

We have outlined multiple steps, starting from early planning, CAD design methods, material selection, and post-processing, all to guide our use-based process that combines clinical, technical, and engineering expertise. Our approach not only enables the design and testing of patient-specific cardiac models but also creates a platform for experiential education, real-time feedback, and accelerated prototyping, all within a clinically integrated environment.

Given individual abilities and variations in institutional practice, we outline a bench-to-bedside proposal including processes and individual expertise needed to perform key steps to successfully translate clinical dilemmas into tangible solutions. Furthermore, this highlights the benefit of single turnkey opportunities at larger institutions, like spoke-and-hub models for patient care, but focused on biomedical engineering and translational clinical science—aligning production expertise with clinical know-how.

### 3.4. Future Directions

To scale this model further, future efforts must focus on expanding access to these technologies, optimizing material properties for physiological realism, and ensuring alignment with clinical implementation and regulatory pathways. By fostering cross-disciplinary collaboration and embedding innovation into both education and practice, we are shaping a future where custom medical solutions are not just possible but expected.

Current trends in cardiovascular research have recently highlighted novel energetic and myocardial mechanical properties—using physiological targets rather than imaging or lab-based targets—that highlight the ability to understand heart failure progression at a granular level. Integration of these data may allow for better modeling and Insilco reproduction of real-world physiology [19].

In summary, this study highlights the feasibility and utility of combining digital anatomy printing and silicone molding to produce anatomically accurate and mechanically relevant left ventricular models. This approach lays the groundwork for future studies in device testing, simulation-based training, and the clinical translation of patient-specific cardiac solutions.

## 4. Conclusions

3D anatomical modeling is rapidly advancing from a static visualization tool to a dynamic platform for functional testing and translational development of cardiac therapeutics. This evolution reflects a broader shift in healthcare innovation, one that fuses engineering, clinical insight, and material science into a cohesive ecosystem for medical advancement.

## Data Availability

Dataset available on reasonable request from the authors.
